# A heparin–rosuvastatin-loaded P(LLA-CL) nanofiber-covered stent inhibits inflammatory smooth-muscle cell viability to reduce in-stent stenosis and thrombosis

**DOI:** 10.1186/s12951-021-00867-8

**Published:** 2021-04-29

**Authors:** Yingjun Liu, Peixi Liu, Yaying Song, Sichen Li, Yuan Shi, Kai Quan, Guo Yu, Peiliang Li, Qingzhu An, Wei Zhu

**Affiliations:** 1 Department of Neurosurgery, Huashan Hospital, Shanghai Medical College, Fudan University, Shanghai, China; 2 Neurosurgical Institute of Fudan University, Shanghai, China; 3 Shanghai Clinical Medical Center of Neurosurgery, Shanghai, China; 4 Shanghai Key Laboratory of Brain Function and Restoration and Neural Regeneration, Shanghai, China; 5 Department of Neurology, Renji Hospital of Shanghai Jiao Tong University, Shanghai, China; 6 Neuroscience and Neuroengineering Research Center, Med-X Research Institute and School of Biomedical Engineering, Shanghai Jiao Tong University, Shanghai, China

**Keywords:** Rosuvastatin, Intracranial aneurysm, Late thrombosis, Long-term arterial stenosis, Nanofiber-covered stent

## Abstract

**Background:**

An endovascular covered-stent has unique advantages in treating complex intracranial aneurysms; however, in-stent stenosis and late thrombosis have become the main factors affecting the efficacy of covered-stent treatment. Smooth-muscle-cell phenotypic modulation plays an important role in late in-stent stenosis and thrombosis. Here, we determined the efficacy of using covered stents loaded with drugs to inhibit smooth-muscle-cell phenotypic modulation and potentially lower the incidence of long-term complications.

**Methods:**

Nanofiber-covered stents were prepared using coaxial electrospinning, with the core solution prepared with 15% heparin and 20 µM rosuvastatin solution (400: 100 µL), and the shell solution prepared with 120 mg/mL hexafluoroisopropanol. We established a rabbit carotid-artery aneurysm model, which was treated with covered stents. Angiography and histology were performed to evaluate the therapeutic efficacy and incidence rate of in-stent stenosis and thrombosis. Phenotype, function, and inflammatory factors of smooth-muscle cells were studied to explore the mechanism of rosuvastatin action in smooth-muscle cells.

**Result:**

Heparin–rosuvastatin-loaded nanofiber scaffold mats inhibited the proliferation of synthetic smooth-muscle cells, and the nanofiber-covered stent effectively treated aneurysms in the absence of notable in-stent stenosis. Additionally, in vitro experiments showed that rosuvastatin inhibited the smooth-muscle-cell phenotypic modulation of platelet-derived growth factor-BB induction and decreased synthetic smooth-muscle-cell viability, as well as secretion of inflammatory cytokines.

**Conclusion:**

Rosuvastatin inhibited the abnormal proliferation of synthetic smooth-muscle cells, and heparin–rosuvastatin-loaded covered stents reduced the incidence of stenosis and late thrombosis, thereby improving the healing rates of stents used for aneurysm treatment.

**Graphic abstract:**

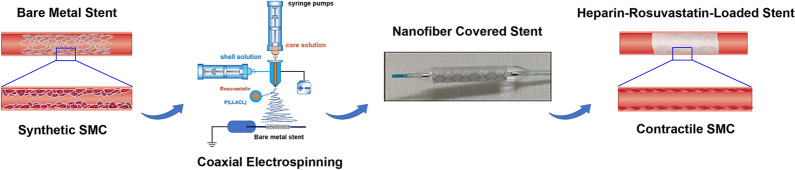

**Supplementary Information:**

The online version contains supplementary material available at 10.1186/s12951-021-00867-8.

## Background

Intracranial aneurysm (IA) is a cerebrovascular disease with an annual incidence of ~ 3% [1] and an annual rupture rate of ~ 1.4% [2], and the mortality rate after aneurysm rupture can be as high as 60% [3]. The International Subarachnoid Aneurysm Trial [4, 5] and the International Study of Unruptured Intracranial Aneurysms [6, 7] showed that interventional therapy results in a lower mortality rate than craniotomy [8]. Although coil implantation is the basic method of IA treatment [9], it is unsuitable for complex IAs. Typical stents implanted to treat IAs currently include flow-diverter devices [10] and the Willis covered stent [11, 12]. Long-term stenosis and late thrombosis can substantially affect the therapeutic effect of the stent; moreover, stents capable of carrying drugs that inhibit cell proliferation may successfully address these issues. Although first-generation drug-eluting stents (DESs) appeared to improve short-term, in-stent stenosis, the ability of drugs to inhibit cell proliferation can cause delayed re-endothelialization. Second-generation DESs improved drug loading and unilaterally inhibited the abnormal proliferation of smooth-muscle cells (SMCs) without affecting endothelial-cell (EC) functions, which successfully addressed the delayed re-endothelialization problem.

DES polymers, such as polytetrafluoroethylene (PTFE) and polyethylene terephthalate, can aggravate the natural inflammatory response [13] by promoting activation of inflammatory SMCs, thereby leading to in-stent stenosis. Although PTFE shows excellent performance in wide arteries, it is unsuitable for arteries narrower than 6 mm in diameter [14]. To address this problem, Stack et al. [15, 16] developed a biodegradable scaffold with poly-l-lactide (PLLA), where the biodegradable membrane decomposes into harmless molecules [17]. PLLA-*co*-caprolactone [P(LLA-CL)] is a biodegradable copolymer that can stably combine with drugs and shows excellent mechanical properties [18, 19]. Additionally, the biodegradation rate of P(LLA-CL) can be controlled by adjusting the molar ratio of PLLA in the copolymer, which makes it more suitable as a stent-coating material.

Although a previous study showed that stent-induced platelet and EC activation could increase the risk of stenosis [20], the anticoagulant heparin could reduce thrombosis and prolong blood clotting after endothelial injury. Because rosuvastatin could promote EC proliferation and inhibit inflammatory-SMC proliferation [21], the combination of heparin and rosuvastatin might represent an effective drug coating for application in next-generation stents. Therefore, in this study, we prepared a nanofiber-covered stent with a stable, biodegradable copolymer and loaded with both heparin and rosuvastatin using coaxial electrospinning.

## Results

### Fabrication and structural characteristics of nanofiber scaffold mats and covered stents

**Fig. 1:**
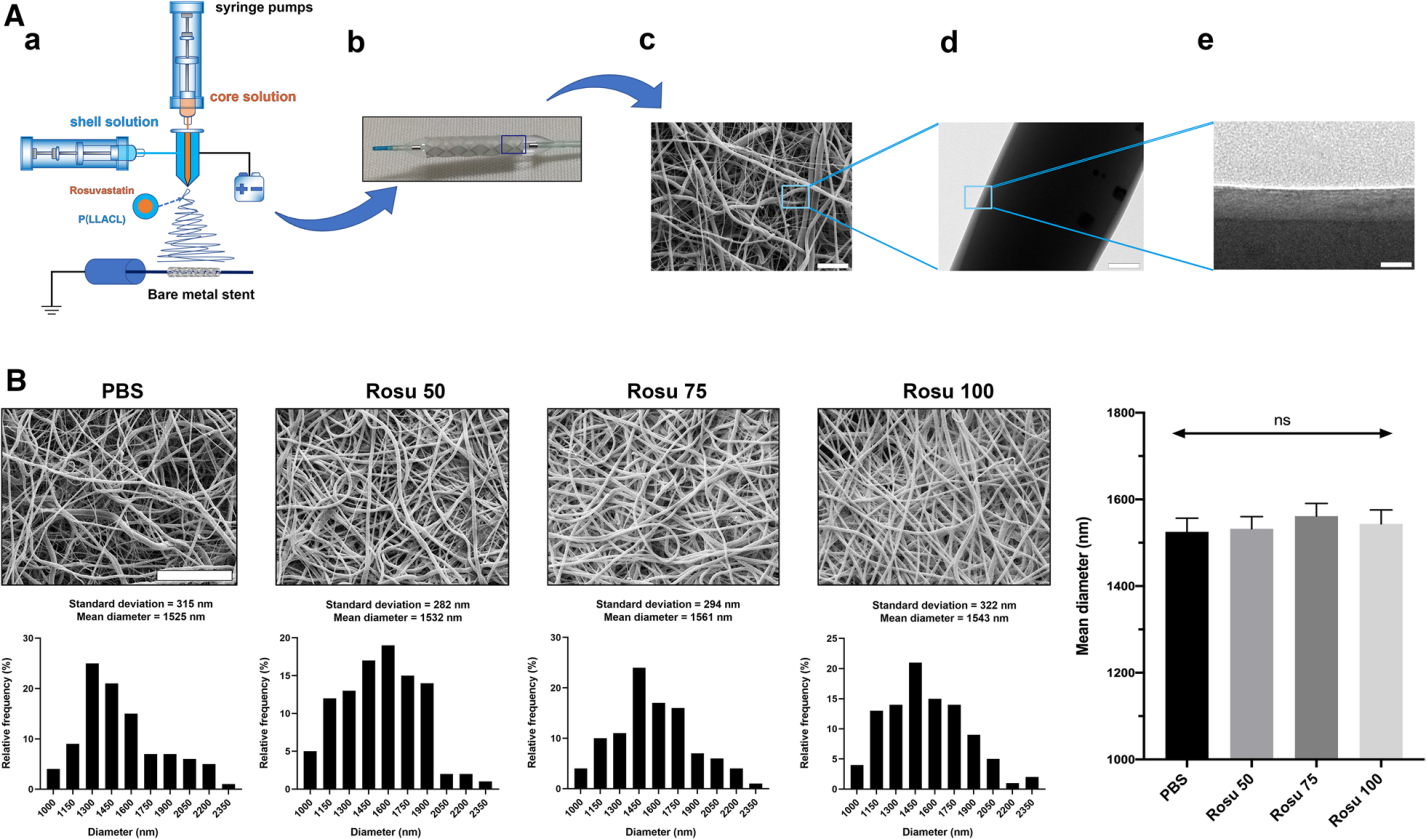
Nanofiber characterizations and mechanical properties. **A** Characteristics of the nanofiber-covered stents. (a) Schematic diagram of stent-graft fabrication, (b) the nanofiber-covered stent, and (c) SEM images of the nanofiber mats. Scale bar, 10 µm. (d,e) TEM image of the core–shell structure. Scale bar, 250 nm and 20 nm. **B** Diameter distribution of the nanofiber mats. Scale bar, 25 µm

Nanofiber scaffold mats were fabricated using coaxial electrospinning (Fig. 1Aa) and then used to completely wrap the stent grafts and form covered stents (Fig. 1Ab). Magnified images clearly show that the nanofiber scaffold mats comprised fibrous structures and showed no structural dissolution at 37 °C (Fig. 1Ac). Transmission electron microscopy (TEM) of the morphology of the nanofiber scaffold mats revealed a light and thin shell with clear edges and a dark drug-loaded core with no aggregation or discontinuity (Fig. 1Ad, e). The core solution was prepared with 15% heparin and 20 µM rosuvastatin solution at volumetric ratios of heparin:rosuvastatin of 450:50 (µL), 425:75 (µL), and 400:100 (µL). The control group used phosphate-buffered saline (PBS) solution instead of heparin and rosuvastatin. Figure 1B shows the mean diameters of the four groups of nanofiber scaffold mats. Rosu 50 (450:50 (µL)), Rosu 75 (425:75 (µL)), and Rosu 100 (400:100 (µL)) core–shell fibers were slightly thicker than those of the control fiber; however, there were no statistical difference between their diameters.

### Effects of heparin–rosuvastatin-loaded nanofiber scaffold mats on synthetic SMCs

**Fig. 2:**
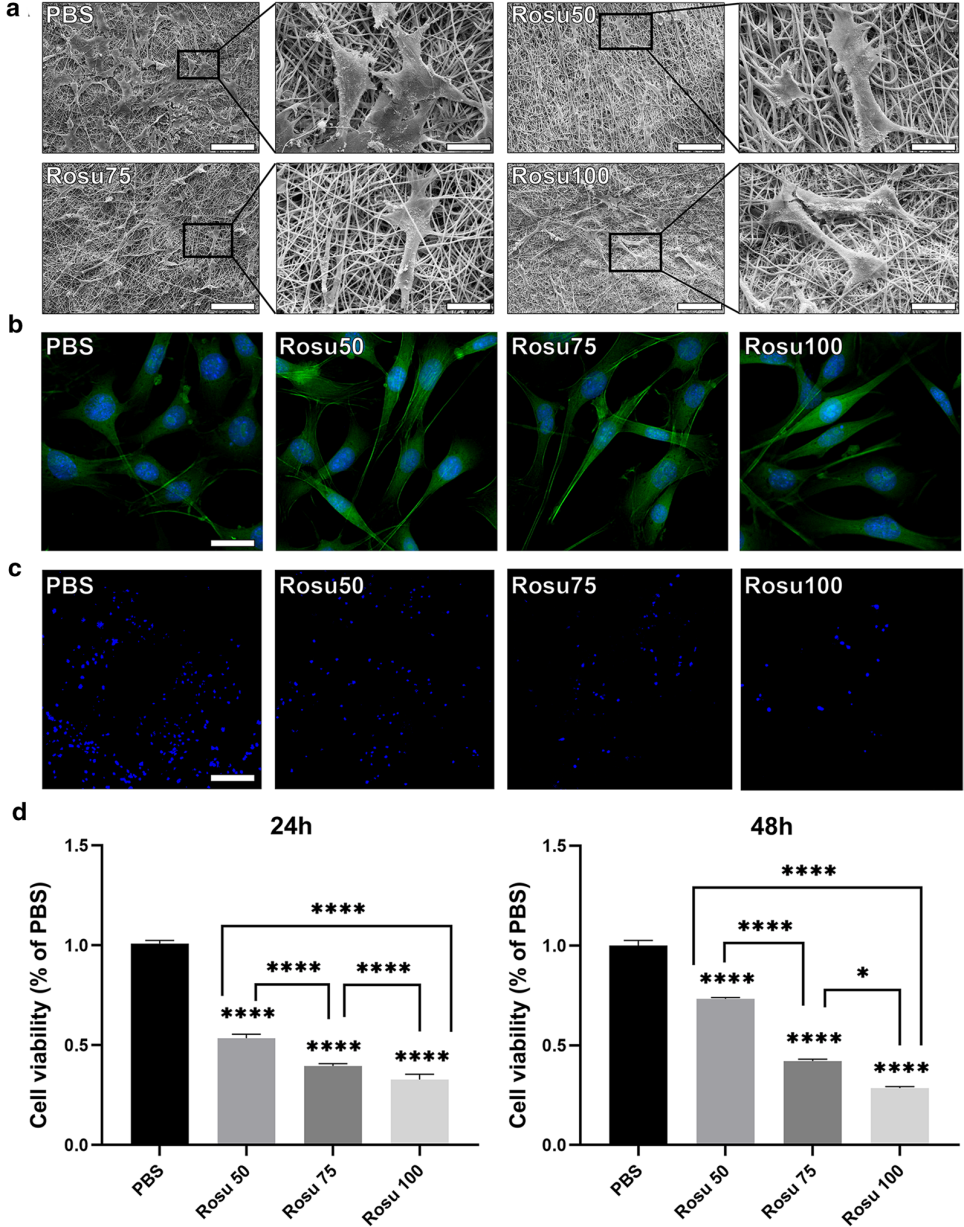
Viability and morphology of attached SMCs. **a** SEM images of synthetic SMCs attached to control, Rosu 50, Rosu 75, and Rosu 100 nanofiber mats. Scale bar, 100 µm. Corresponding magnified image. Scale bar, 25 µm. **b** Phalloidin-labeled SMCs attached to nanofiber mats. Scale bar, 30 µm. **c** Hoechst-33342-labeled SMCs attached to nanofiber mats. Scale bar, 150 µm. **d** Bar graph showing the viabilities of SMCs attached to nanofiber mats after 24 h and 48 h

**Fig. 3:**
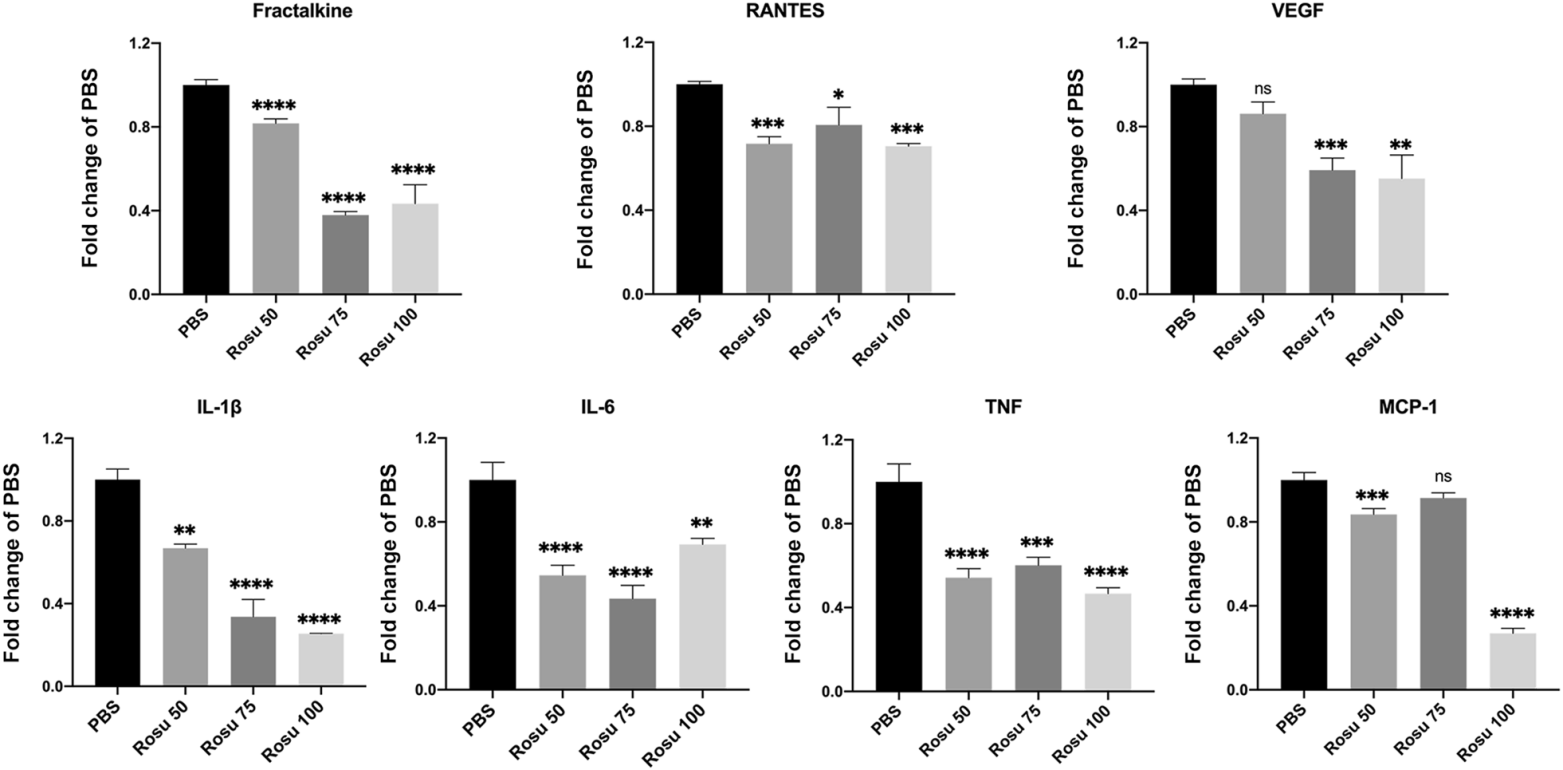
Cytokine secretion by attached SMCs. Bar graph shows the levels of inflammatory factors secreted by SMCs attached to nanofiber mats according to MILLIPLEX MAP rat cytokine/chemokine factor panel. Data represent the mean ± SD (*n* = 3/group). *p < 0.05, ****p < 0.0001

SMCs were stimulated with platelet-derived growth factor (PDGF)-BB for 24 h. Scanning electron microscopy (SEM) indicated that the SMCs appeared as flat triangles and less-contracted spindles in the control group. In the presence of increasing rosuvastatin concentrations, we observed a spindle-like cell morphology in the Rosu 50 and Rosu 75 groups, and the number of flat triangular cells decreased. In the Rosu 100 group, cells were slender and spindle-like, and their morphological changes were more obvious than those of control cells (Fig. 2a). Phalloidin staining showed that increasing rosuvastatin concentration promoted a gradual increase in the spindle-like morphology (Fig. 2b), and Hoechst-33342 staining showed that an increased rosuvastatin ratio also decreased the number of attached, synthetic SMCs (Fig. 2c). Cell counting kit-8 assays to quantify the number of synthetic SMCs attached to the nanofiber scaffold mats revealed that after 24 h and 48 h of culture, the highest synthetic SMC proliferation occurred in the control group, with this activity decreasing at higher rosuvastatin concentrations, thereby showing a negative correlation (Fig. 2d). Moreover, detection of secreted inflammatory factors indicated significant decreases in the Rosu 100 group relative to the control (Fig. 3).

### Balloon-expansion assay

**Fig. 4:**
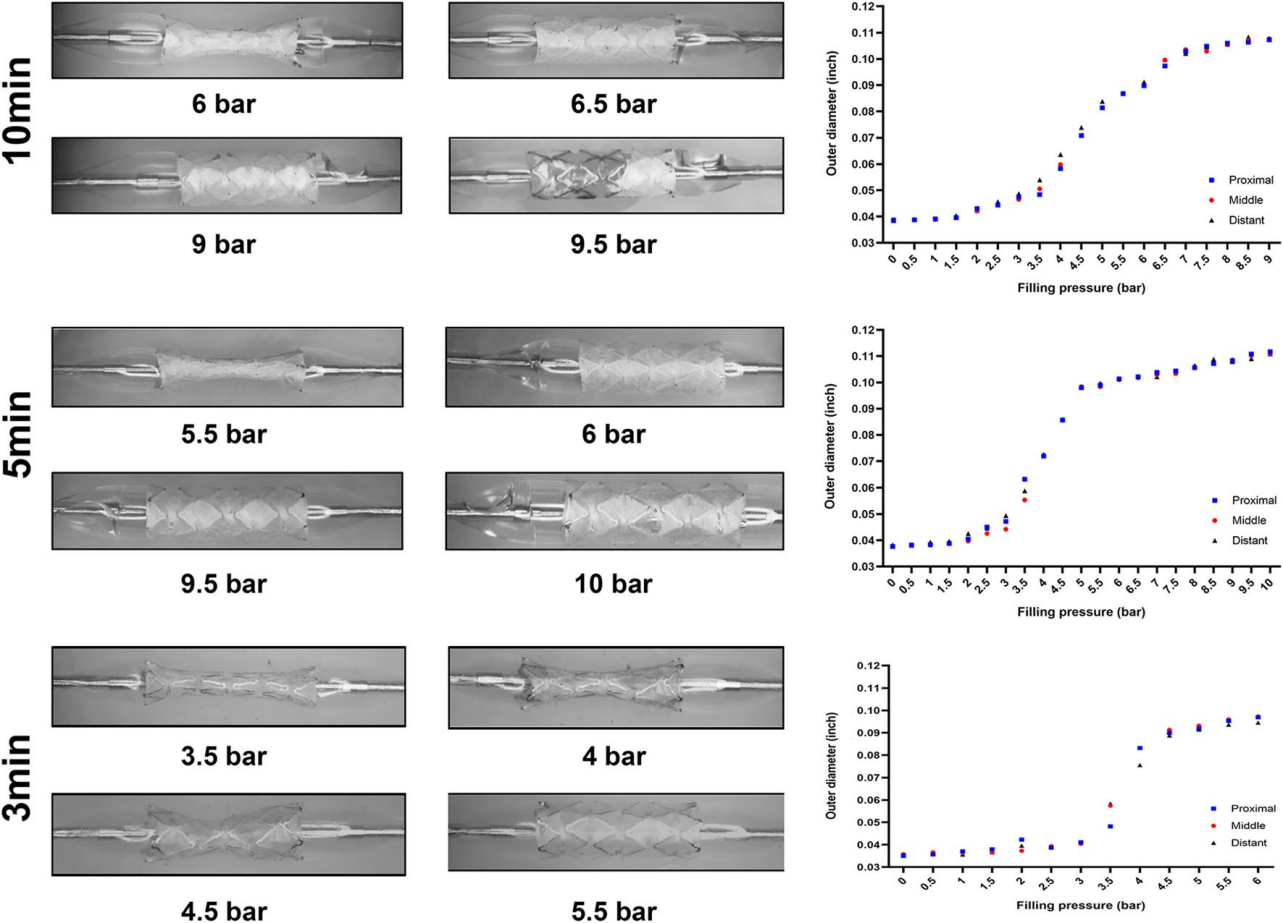
Images and corresponding scatter charts for balloon-expansion experiments on covered stents fabricated with nanofiber scaffolds spun for different times (3, 5, and 10 min)

Based on these findings, we preliminarily chose Rosu 100 as the most suitable volumetric ratio and fabricated nanofibers on stents for 3 min, 5 min, and 10 min in order to test the different thicknesses. Figure 4 shows the results of the balloon-expansion experiment. For nanofibers spun for 10 min, the proximal and distal ends of the stent began to expand at 4 bar; however, the expanded balloon could not drive the middle segment to expand, leading to a dog-bone-like stent graft. At 6.5 bar, the stent opened in pulsations, and at 9 bar, we observed no translucent phenomenon. The covered nanofiber scaffold mats retained their thickness and remained wrapped around the stent, thereby limiting stent expansion. Simultaneously, we observed tiny cracks on the covered mats, and at 9.5 bar, the balloon ruptured and leaked. For nanofibers spun for 5 min, the proximal and distal ends of the stent began to expand at 4 bar. When the filling pressure reached 6 bar, the stent expanded in pulsations, and when the pressure reached 9.5 bar and 10 bar, fine cracks were observed, although the nanofiber mats appeared transparent and continued to cover the stent. For nanofibers spun for 3 min, the proximal and distal ends of the stent began to expand at 3.5 bar, the dog-bone shape disappeared at 5 bar, and the stent expanded completely, which differed from the other two nanofiber mats. Upon complete expansion of the stent, the mats were clearly wrapped around the stent, and the stent expanded smoothly without sudden expansion. When the filling pressure reached 6 bar, the stent was completely expanded, and the mat remained intact. The scatter chart prepared according to data obtained for the stent outer diameter showed that the outer diameter of the stent graft could be expanded by changing the pressure. The optimal pad thickness was obtained for the nanofiber scaffolds spun for 3 min; therefore, we performed subsequent experiments using these stents.

### Establishment of the rabbit aneurysm model, stent implantation, and short- and long-term follow-ups

**Fig. 5:**
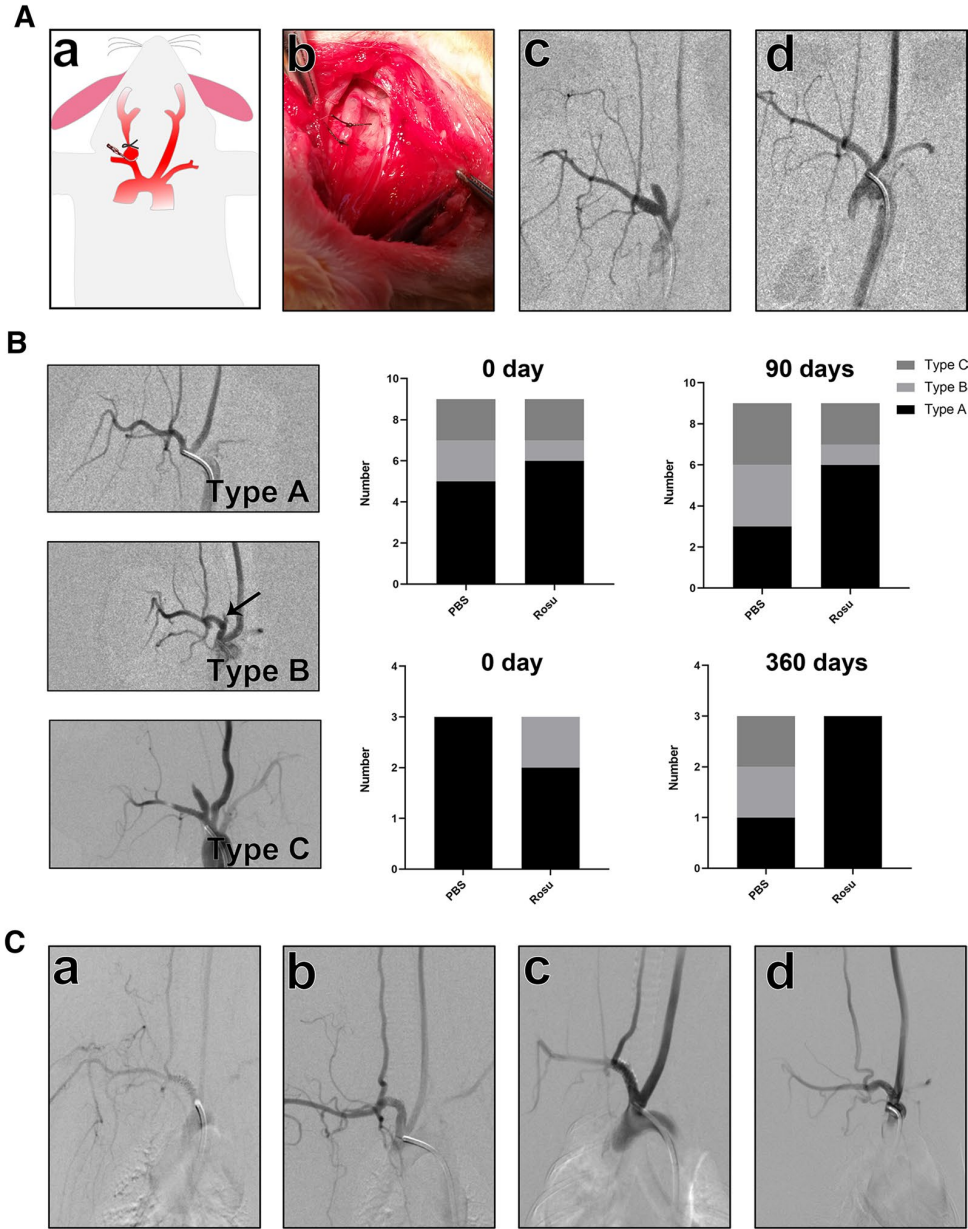
Establishment of the rabbit aneurysm model and short- and long-term follow-ups. **A** Initiation of the rabbit aneurysm model and stent implantation: (a) Schematic diagram of aneurysm initiation, (b) photograph of the induced aneurysm, (c) angiography of the aneurysm after 30 days, and (d) angiography of aneurysm occlusion after stent implantation. **B** Angiography illustrations of type A, B, and C aneurysms at follow-up. Bar graph shows the number of different types in control and Rosu 100 groups immediately after implantation and at 3- and 12-month follow-ups. **C** Angiography of the parent artery at (a,b)3- and (c,d)12-month follow-ups in the control and Rosu 100 groups

Aneurysm in rabbits was induced by injecting porcine pancreatic elastase into the right common carotid artery (CCA) of New Zealand white rabbits (Fig. 5Aa). After induction, the right CCA was larger than a normal blood vessel and formed a longitudinal aneurysm body, with the right subclavian artery representing the parent artery (Fig. 5Ab). Digital subtraction angiography (DSA) was performed 30 days after aneurysm induction. Angiography showed that aneurysms were induced (Fig. 5Ac) and that the covered stent was delivered to the parent artery to cover the aneurysm neck. With the expansion of the covered stent, the aneurysm body completely disappeared in the DSA images (Fig. 5Ad).

After stent implantation, aneurysms were divided into three grades and used to evaluate the therapeutic effect during follow-up. Table 1 shows the grading standards, and Fig. 5B shows the angiography images of the three grades. Most of the stents achieved good coverage on immediate postoperative angiography after stent implantation, and there was no significant difference between the control and Rosu 100 groups. However, at the 3-month (short-term) follow-up in the control group, the number of type A grades decreased by 40%, whereas types B and C grades both increased by 50%. In contrast, the number of each grade in the Rosu 100 group did not change, and the stent did not promote thrombosis in the aneurysms. At the 12-month (long-term) follow-up, the number of type A grades in the control group decreased by 33%, and the number of types B and C grades both increased by 100%. The Rosu 100 group showed one case of slight blood leakage into the aneurysm cavity after treatment, which had been rated as type B grade after the operation. At the 12-month follow-up, the aneurysm with the original type B grade was no longer developed, and all three aneurysms were cured, reaching type A grade (Fig. 5B). We further analyzed the changes in the parent artery after stent implantation, finding that in both the short- and long-term follow-ups, there was no obvious stenosis in the parent arteries (Fig. 5C).

**Table 1:** Aneurysm classification

Type	Description
Type A	Aneurysm shows no recanalization or bleeding after stent implantation and follow-up
Type B	Dog-ear-shaped aneurysm showing mild blood leakage after stent implantation and follow-up
Type C	Aneurysm has obviously not healed and shows recanalization after stent implantation and follow-up

### Histologic analysis

**Fig. 6:**
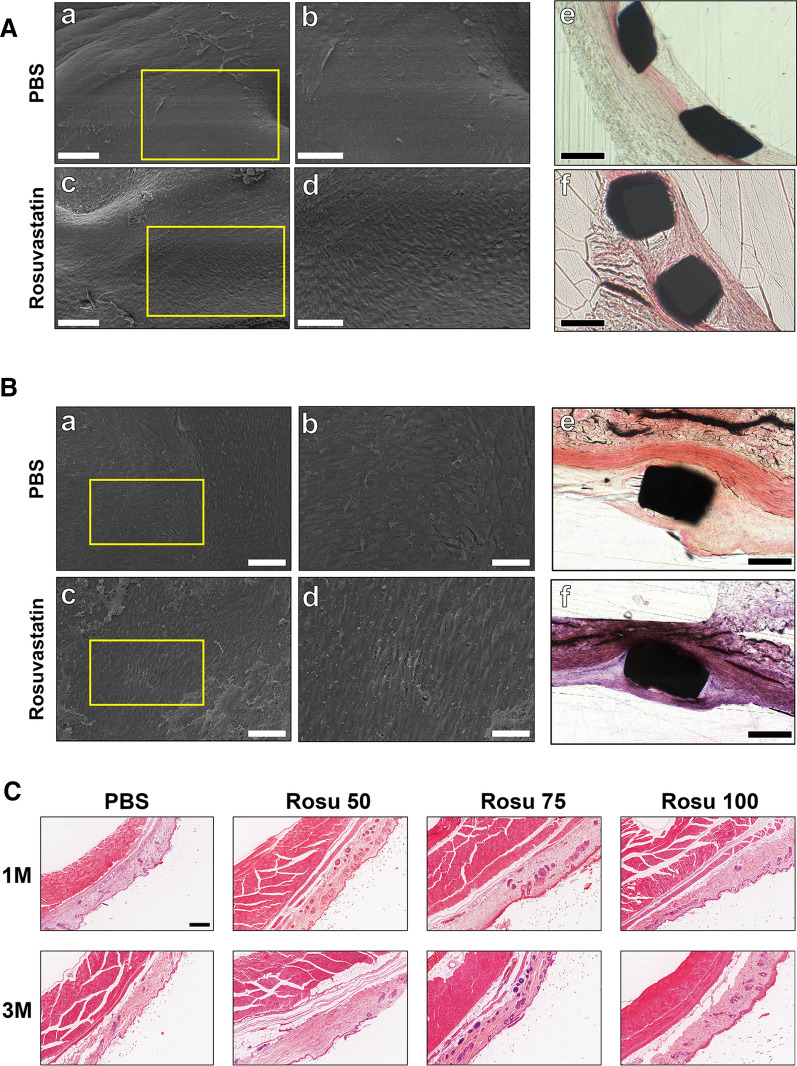
SEM, histology, and toxicity of the nanofiber scaffold mats. **A** SEM and H&E-stained vascular-section images showing endothelialization of the covered stent at 3 months. **B** SEM and H&E-stained vascular-section images showing endothelialization of the covered stent at 12 months. Scale bars: (a, c) 150 µm and (b, d, e, f) 100 µm. **C** H&E-stained vascular-section images showing the effects of control, Rosu 50, Rosu 75, and Rosu 100 nanofiber mats implanted under abdominal subcutaneous tissue for 1 and 3 months. Scale bar, 250 µm

SEM and hematoxylin and eosin (H&E) staining showed better endothelial coverage in the Rosu 100 group relative to the control at the 3-month follow-up (Fig. 6Aa–f). At the long-term follow-up (Fig. 6B), the control and Rosu 100 groups both showed similar endothelial coverage. Additionally, toxicity experiments revealed no inflammatory reactions in H&E staining at either 1 or 3 months (Fig. 6C).

### Establishment of a PDGF-BB-induced synthetic SMC model and the viability of rosuvastatin-treated SMCs

Additional file 1: Figure S1A shows that 10 ng/mL PDGF-BB significantly increased SMC viability at 24 h (*p* < 0.0001). Moreover, we found that SMC proliferation increased along with elevated PDGF-BB concentration; however, at 1000 ng/mL, the proliferative activity decreased, suggesting an adverse reaction. Additional file 1: Figure S1B shows the results of rosuvastatin treatment of contractile SMCs. Although contractile-SMC proliferation was not significantly inhibited at low rosuvastatin concentrations (*p* > 0.05), it was significantly inhibited at 100 µM (*p* < 0.0001). To determine the effective inhibitory rosuvastatin concentration in the presence of PDGF-BB, we treated SMCs with 10 ng/mL of PDGF-BB and varied the rosuvastatin concentration for a 24-h incubation. CCK-8 assays showed that 5 μM rosuvastatin inhibited PDGF-BB-induced synthetic-SMC proliferation (*p* < 0.05), with the inhibitory effect significant at 10 μM (*p* < 0.0001) and strengthened along with increasing rosuvastatin concentrations (Additional file 1: Figure S1C). Furthermore, flow cytometric results showed that 10 µM rosuvastatin calcium did not cause abnormal apoptosis in contractile SMCs (Additional file 1: Figure S2); therefore, we used 10 µM of rosuvastatin calcium for subsequent experiments.

### SMC morphology, viability, and function

**Fig. 7:**
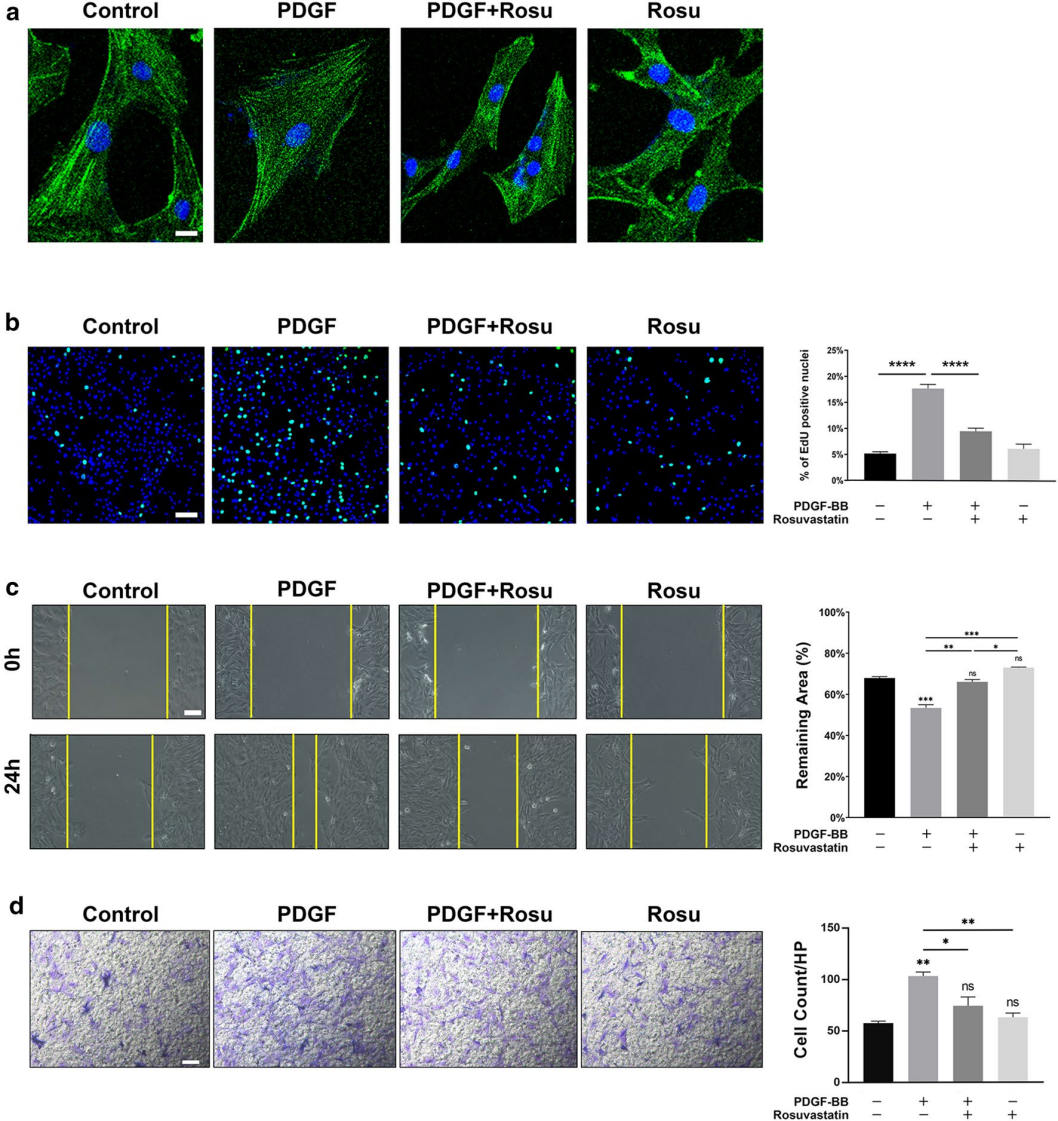
Cell morphology, viability, and function of PDGF-BB- and rosuvastatin-treated SMCs. **a** Cell morphology of control, PDGF, PDGF + Rosu, and Rosu groups. Scale bar, 20 µm. **b** Cell proliferation rate of control, PDGF, PDGF + Rosu, and Rosu groups. Scale bar, 100 µm. Bar graph showing the cell proliferation rate (%). Data represent the mean ± SD (*n* = 3/group). ****p < 0.0001. **c** Wound-healing assays and corresponding representative images of SMC migration in control, PDGF, PDGF + Rosu, and Rosu groups according to scratch assay. Scale bar, 100 µm. Data represent the mean ± SD (*n* = 3/group). *p < 0.05, **p < 0.01, ***p < 0.001. **d** Transwell assays and corresponding representative images of crystal violet-stained invasive cells in the lower chamber. Scale bar, 100 µm. Data represent the mean ± SD (*n* = 3/group). *p < 0.05, **p < 0.01

Phalloidin staining to evaluate cell morphology in the control and Rosu groups showed that the cells remained spindle-shaped, whereas cells in the PDGF group appeared flat and triangular. However, given that PDGF-BB-induced alteration of SMC morphology was significantly inhibited by rosuvastatin, the cell morphology in the PDGF+Rosu group was more spindle-like (Fig. 7a).

EdU staining revealed that after stimulation with 10 ng/mL PDGF-BB for 24 h, the number of EdU-labeled SMCs increased significantly (*p* < 0.0001); however, co-treatment of cells with rosuvastatin significantly decreased the number of EdU-labeled SMCs relative to that observed in the PDGF-BB-induced group in the absence of rosuvastatin (*p* < 0.0001) (Fig. 7b).

For the scratch test, after 24 h of intervention, 67% of the remaining area in the control group remained unhealed, whereas only 53% remained unhealed in the PDGF group (*p* < 0.001). Although the residual area in the PDGF + Rosu group was 66%, which differed significantly from that in the PDGF group (*p* < 0.01), the residual area in the Rosu group was not notably different from that in the control group (Fig. 7c).

Transwell assay results showed that although cell penetration in the Rosu groups was weak relative to that observed in the control group, the number of cells that had penetrated the PDGF group increased significantly (*p* < 0.01). Moreover, although SMC migration improved substantially following PDGF-BB stimulation, the number of penetrating cells decreased after rosuvastatin treatment, which differed significantly from the trend observed in the PDGF group (Fig. 7d).

### Effects of rosuvastatin on SMC phenotype and inflammatory factors

**Fig. 8:**
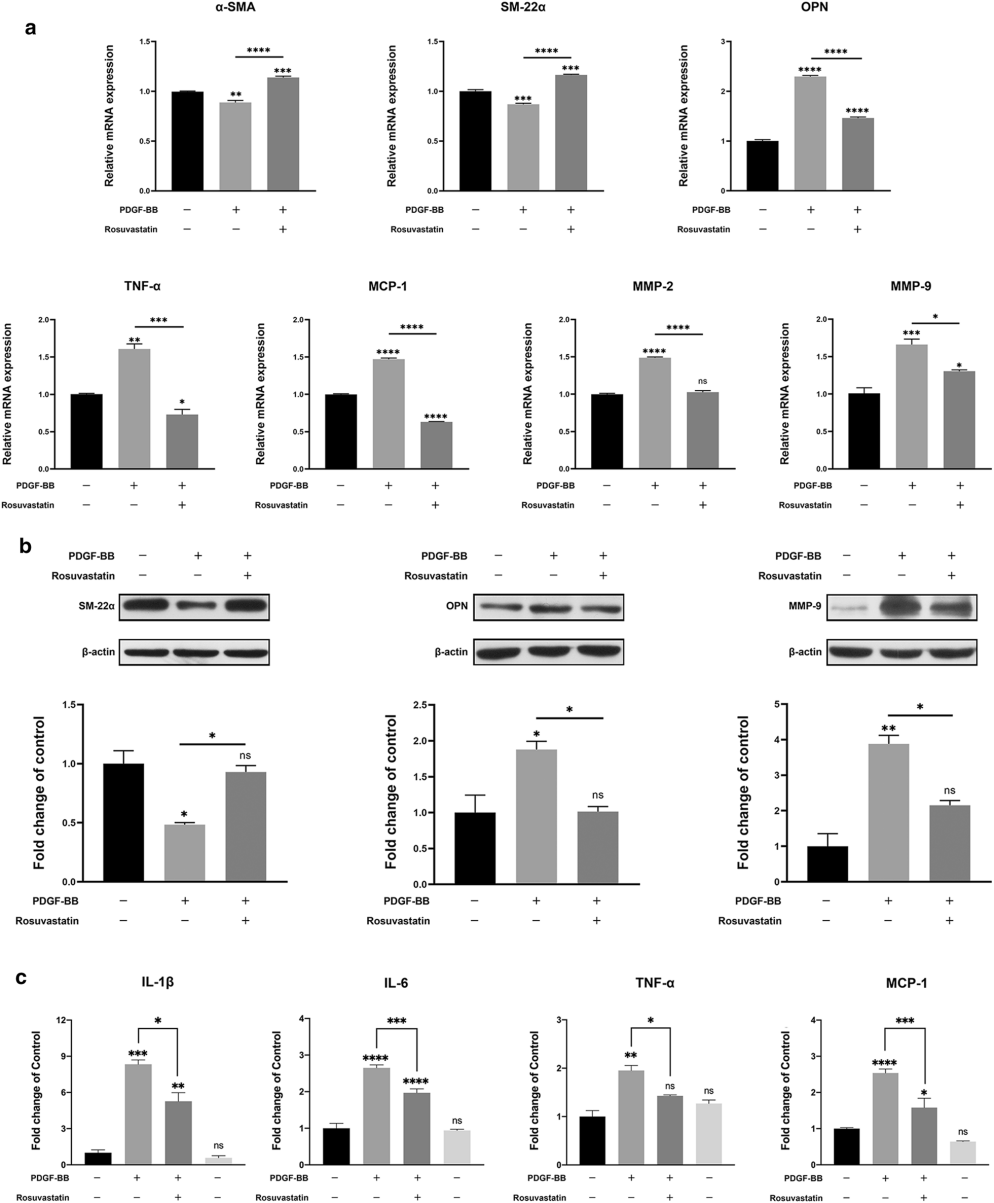
Effects of PDGF-BB- and rosuvastatin-treated SMCs on phenotype markers and inflammatory cytokines. **a** Fold changes in mRNA levels of *α-SMA*, *SM22-α, OPN*, and inflammatory cytokines. Data represent the mean ± SD (*n* = 3/group). *p < 0.05, **p < 0.01, ***p < 0.001, ****p < 0.0001. **b** Western blot analysis of SM22-α, OPN, and MMP-9 levels. Data represent the mean ± SD (*n* = 3/group). *p < 0.05, **p < 0.01. **c** Inflammatory cytokines analyzed using MILLIPLEX MAP rat cytokine/chemokine factor panel. Data represent the mean ± SD (*n* = 3/group). *p < 0.05, **p < 0.01, ***p < 0.001, ****p < 0.0001

Polymerase chain reaction (PCR) results showed that the expression of contractile phenotypic markers *α-*smooth muscle actin (*SMA*) and *SM22-α* was lower in the PDGF group than in the control group; however, after rosuvastatin treatment, *α-SMA* and *SM22-α* expression increased. Subsequent evaluation of osteopontin (*OPN*) expression for reverse verification indicated its upregulation in the PDGF group and obvious attenuation following rosuvastatin treatment. Moreover, expression levels of tumor necrosis factor (*TNF*)*-α*, monocyte chemoattractant protein (*MCP*)*-1*, matrix metalloproteinase (*MMP*)*-2*, and *MMP-9* also decreased substantially following rosuvastatin treatment (Fig. 8a).

Next, we detected protein levels of phenotype markers and inflammatory factors in PDGF-BB-induced SMCs using western blotting. The results showed that SM22-α and OPN were notably downregulated and upregulated after PDGF-BB stimulation (p < 0.05), respectively, and that the protein expression of MMP-9 was also notably increased after PDGF-BB stimulation (p < 0.01). With the addition of rosuvastatin, the SM22-α expression increased (p < 0.05), OPN and MMP-9 expressions decreased (p < 0.05), respectively (Fig. 8b).

Subsequently, we evaluated levels of secreted inflammatory factors following PDGF-BB stimulation, finding elevated levels in all analyzed factors, with interleukin (IL)-1β secretion increased fourfold relative to levels secreted by the control group. Compared with the PDGF group, rosuvastatin-treated SMCs showed significantly decreased secretion of all inflammatory factors (Fig. 8c).

## Discussion

To prevent stent stenosis and thrombosis, drugs loaded onto covered-stent grafts should be able to regulate cell proliferation, inflammatory reactions, and thrombosis. After stent implantation, effective anticoagulation and inhibition of platelet adhesion are the first steps. Heparin plays a major role in anticoagulation therapy, and although heparin coating reduced the thrombosis rate in animal experiments [22, 23], it could not effectively improve late vascular patency and neointimal hyperplasia [24], suggesting that promoting the proliferative ability of ECs is crucial. In our previous study, we found that rosuvastatin- and heparin-coated stents effectively promoted early endothelialization and provided a basis for reducing long-term complications [25]. Early endothelialization is the key to preventing in-stent stenosis and late thrombosis [26, 27]. Willis covered stents are widely used in the treatment of complex aneurysms; however, although expanded PTFE reduces the incidence of early stent-graft stenosis, it is also prone to delayed re-endothelialization [28]. Non-degradable polymers that remain in arteries can continue to cause local inflammatory reactions [29]. In patients treated with PTFE-covered stents, the incidence of nonfatal myocardial infarction is higher than that in patients treated with a bare metal stent [30]. Although PLLA is a biodegradable synthetic polymer, it shows poor flexibility; however, blending PLLA with a more elastic polymer is an effective method of improving its mechanical properties [31, 32]. Previous studies show that the P(LLA-CL) nanofiber membrane shows good EC adhesion [33]; therefore, we chose P(LLA-CL) as a foundation for the development of nanofiber scaffolds. Because the cover mats of the Willis stent graft are loosely sutured to the stent [34], the nanofibers might be damaged during placement [35].

Electrospinning can prepare nanometer-scale-diameter structures and fabricate complex and diverse structures [36] while maintaining good material properties [37, 38]. Electrospun polymers can deliver a variety of therapeutic agents and demonstrate broad application value in biomedicine [39]. Additionally, studies indicate that nanofiber scaffolds prepared by coaxial electrospinning are less likely to be damaged and that coaxial electrospinning can not only load drugs evenly onto polymers but also facilitate drug release. Furthermore, coaxial electrospinning can prepare shell–core structures [33, 40], thereby allowing polymer drug loading, which is beneficial to the combination of drugs and polymers. Uniform drug loading can provide a good foundation for drug release, and sustained drug release can provide a stable therapeutic effect. Moreover, in cerebral arteries, the stent-graft needs to be miniaturized and show high flexibility and maneuverability in order to smoothly navigate to the target artery [41]. Therefore, we used an Apollo stent made of 316L stainless steel, which shows sufficient supporting force and a special spatial structure capable of providing effective toughness during stent expansion, which is critical, as excessive pressure during balloon expansion can lead to thromboembolism, vascular dissection, or rupture [42]. The expansion pressure of a bare Apollo stent is only 6 bar. In the present study, nanofiber spinning for 3 min resulted in membrane stability at 6 bar, indicating that a 3-min-thick membrane was more suitable to enable complete stent opening under low pressures without affecting the pressure of the Apollo stent itself. Therefore, we adopted the balloon catheter expandable stent and set the membrane thickness at 3 min.

The elastase-induced aneurysm in rabbits represents a mature aneurysm model [43]; however, different animal models have different research objectives. In the cardiovascular system, the porcine coronary artery model can be used to evaluate the risk of stent stenosis. The tendency of thrombosis and neointimal formation in the porcine coronary system is similar to that in humans, which is especially helpful in evaluating arterial stenosis after stent implantation [44, 45]. However, porcine coronary artery is unsuitable for simulating the environment of small arteries. In addition to porcine and rodent models, the atherosclerosis model of New Zealand white rabbits can also be used to evaluate stent stenosis. Stents implanted in New Zealand white rabbits fed a high-fat diet to evaluate in-stent stenosis revealed this in-stent stenosis model as more suitable for observing the drug mechanism as compared with the porcine model [46, 47]. Additionally, use of a rabbit CCA model to evaluate in-stent stenosis is an emerging method, with studies reporting no differences from traditional methods [48].

The traditional flow-diverter device shows only 56% occlusion at the 3-month follow-up, and it takes 12 months to reach 95% occlusion [49]. In the present study, the aneurysm was immediately excluded from the blood flow after placement of the covered stent, and the aneurysm was no longer visible. Additionally, a study showed that mild endoleaks following implantation of a covered stent could eventually be occluded [35]. In our long-term follow-up, a type C aneurysm sprang a leak, and the aneurysm remained visible after stent implantation; however, after treatment with the Rosu 100-covered stent for 12 months, the aneurysm was no longer visible. Moreover, SEM showed that the endothelial coverage of the aneurysm neck was intact, whereas in the control group that was originally satisfactory after the treatment, only one case did not leak or recanalize at the 12-month follow-up, suggesting that the heparin and rosuvastatin loads showed therapeutic effects and a stable aneurysm-cure rate.

After vascular injury, SMCs can transform from a resting contractile phenotype into a proinflammatory, de-differentiated one (also named synthetic type). The phenotypic regulation of vascular (V)SMCs, which describes SMC differentiation, was first conceptualized by Chamley-Campbell et al. [50]. When the differentiated phenotype is modulated relative to the de-differentiated one, cell morphology changes accordingly. The contractile SMCs appeared as slender and spindle-like, whereas proinflammatory SMCs appeared as flat triangles. Upon SMC transformation to the proinflammatory phenotype, inflammatory cytokines are produced and inflammatory cell markers are expressed [51, 52], with these processes involving the participation of multiple cytokines [53]. PDGF-BB is a key factor affecting the phenotypic modulation of SMCs [54, 55] and promotes changes in SMC morphology from spindle-shaped to flat triangular or oblique squares [56]. Inhibition of PDGF-BB activity increases the concentrations of the contractile-type markers, α-SMA and SM-22α [57]. Moreover, a study showed that rosuvastatin exerted pleiotropic effects and reduced SMC phenotypic modulation [58]. In the present study, we used PDGF-BB to construct a model of inflammatory SMCs in order to simulate the cytokines released by endothelial injury and subsequent modulation of the SMC phenotype. We found that PDGF-BB stimulated SMC proliferation and migration and that elevated PDGF-BB concentrations substantially increased *OPN* expression while reducing the expression of phenotypic markers of contractile SMCs. These findings suggested that the cytokines released after endothelial damage could regulate SMC phenotype. On the nanofiber mats, we observed that increased rosuvastatin concentration reduced the number of PDGF-BB-stimulated SMCs and gradually restored their shape to spindle-like. These results indicated that the rosuvastatin-loaded nanofiber functioned by inhibiting the abnormal proliferation of SMCs.

Statins exert a protective effect on SMCs and can regulate multiple pathways to inhibit their phenotypic modulation [59, 60]. Previous reports indicate that statins can inhibit the transduction of PDGF-BB on SMCs by blocking the G0/G1 cell cycle and the PDGF receptor β-Akt signaling cascade [61], thereby inhibiting the pathological proliferation and migration of SMCs [62]. Furthermore, pitavastatin attenuates the LR11/uPA system to reduce PDGF-stimulated SMC migration [63], and simvastatin can reduce the PDGF/IL-1-induced expression and secretion of MMP-9 in VSMCs by inhibiting the RhoA/Rho-associated protein kinase signaling pathway [64], thereby reducing transduction of the SMC inflammatory response. Following establishment of the PDGF-BB cell model, we used rosuvastatin to treat SMCs, which inhibited PDGF-BB-mediated proliferation and migration of the inflammatory SMCs. Analysis of the secreted supernatant revealed that the expression of inflammatory factors in rosuvastatin-treated SMCs decreased, which demonstrated that rosuvastatin calcium could effectively regulate the SMC phenotype.

## Conclusions

This study evaluated the application of a nanofiber-covered stent in vivo and its mechanism in vitro. In vitro experiments demonstrated that rosuvastatin inhibited the proliferation and migration of inflammatory SMCs, as well as PDGF-BB-mediated induction of SMCs. Importantly, in vivo studies revealed that the covered stent caused no persistent inflammatory reactions in tissues. We found that the therapeutic effect of the Rosu 100-covered stent was ideal for treating rabbit RCCA aneurysms. These results suggest that drug-loaded covered stents can potentially reduce the risk of late in-stent thrombosis and stenosis.

## Materials and methods

### Fabrication of the nanofiber-covered stent

Nanofiber was fabricated, as previously described [65]. P(LLA-CL) was dissolved in 10 mL of hexafluoroisopropanol to prepare a 120 mg/mL shell solution, and the core solution was prepared with 15% heparin and 20 µM rosuvastatin solution (Sigma-Aldrich, Merck, Germany). The volumetric ratios of heparin:rosuvastatin were 450:50 [450:50 (µL)], 425:75 [425:75 (µL)], and 400:100 [400:100 (µL)], and the control group used PBS to replace heparin and rosuvastatin. The nanofiber mats were labeled as control, Rosu 50, Rosu 75, and Rosu 100 according to the different rosuvastatin volumetric ratios. An Apollo bare metal stent made of 316L stainless steel (MicroPort Co., Ltd., Shanghai, China) was placed 10 cm to 25 cm away from the tip of the syringe pump (KDS 200; KD Scientific, Holliston, MA, USA). The core and shell solutions were then used to fabricate the coaxial nanofiber-covered stents by collecting nanofibers with a rotating Apollo bare metal stent (600 rpm, F = 2.3 mm, and l = 7 mm) at room temperature. The structure of the covered stent was observed using a transmission electron microscope (Hitachi, Tokyo, Japan).

### Measurement of nanofiber diameter

Four groups of sterilized nanofiber scaffolds were soaked in Dulbecco’s modified Eagle medium (DMEM; Hyclone, Provo, Utah) for 24 h in triplicate and observed using a scanning electron microscope (Phenom XL; Phenom-World, Utrecht, The Netherlands). The diameters of 100 fibers, as shown in the SEM images, were measured and recorded.

### Characterization of the nanofiber scaffold mats

Control, Rosu 50, Rosu 75, and Rosu 100 were fumigated with 75% alcohol for 3 days and then sterilized by ultraviolet radiation for 30 min to prepare the sterile scaffold mats. SMCs were stimulated with 10 ng/mL PDGF-BB (PeproTech, Rocky Hill, NJ, USA) for 24 h, after which SMCs were seeded into four groups of nanofiber mats at a density of 2 × 10^4^ cells/well in triplicate. After 24 h and 48 h, the intervention was terminated, glutaraldehyde was added to fix the cells, and the samples were observed using SEM. Three regions of each sample were selected for observation.

### Hoechst staining of cells attached to nanofiber scaffold mats

SMCs were stimulated with 10 ng/mL PDGF-BB for 24 h and then seeded on nanofiber mats at a density of 2 × 10^4^ cells/well in triplicate, followed by culture for 48 h. After fixing the cells with 4% paraformaldehyde (PFA; Sinopharm, Shanghai, China) overnight, nanofiber mats were stained with Hoechst-33342 (Beyotime, Beijing, China) for 5 min according to manufacturer instructions. Samples were photographed using a confocal fluorescence microscope (Carl Zeiss, Oberkochen, Germany). The Hoechst-33342-labeled nuclei were stained blue.

### Analysis of attached-SMC viability

SMCs were pretreated with 10 ng/mL PDGF-BB for 24 h and seeded into four groups of nanofiber mats at a density of 10^4^/well in triplicate. A CCK-8 assay (Dojindo, Kumamoto, Japan) was conducted according to manufacturer instructions to study cell viability on the scaffold mats. The seeded mats were cultured for either 24 h or 48 h, after which 500 µL of CCK-8 staining solution was added to each well, and the mats were incubated in 37℃ for another 2 h to 3 h. The absorbance at 450 nm was measured with a microspectrophotometer (Thermo Fisher Scientific, Waltham, MA, USA).

### Balloon-expansion experiment

The covered stent was connected to a balloon catheter (MicroPort Co., Ltd., Shanghai, China) and gradually filled with 0 bar to 10 bar (0–1000 kPa) of air for expansion. The outer diameters of the proximal, distal, and middle segments of the covered stent were measured and statistically graphed.

### Establishment of the rabbit aneurysm model and stent implantation

The New Zealand white rabbit common-carotid-aneurysm model was described previously [66]. At 30 days after initiating the model, which was sufficient for the aneurysm to mature, DSA was used to observe aneurysm formation. Covered stents were placed into the right subclavian artery and maintained at equal lengths on either side of the aneurysm ostium. According to results of the in vitro assay, Rosu 100 and the control were used for the experiments, with the stents were randomly selected from these two groups. Immediately after the procedure, DSA was used to evaluate blood flow in the aneurysm. Based on the DSA characteristics, therapeutic efficacy was divided into three grades (Table 1).

### Short- and long-term follow-ups

The treated animals were administered aspirin (20 mg/day) 7 days prior to and 14 days after stenting. Therapeutic efficacy was evaluated with DSA at 3 months and 12 months after stenting and divided into three grades. Stent grafts were collected at 3 months and 12 months after stent implantation, and SEM and histology were used to observe endothelialization of the parent artery.

### Histologic analysis

Specimens were fixed with 4% PFA overnight and dehydrated using an alcohol gradient (75, 85, 90, 95, and 100%) for 24 h. The specimens were then soaked in xylene for 4 h and treated with curing monomers I, II, and III for 24 h, after which the specimens were encased in monomers 1 cm to 1.5 cm above the tops of the tissue specimens by adding the curing monomer and using a gas pump in a dryer to prevent bubble formation. The tissue specimens were incubated at 4 °C for 1 week, further incubated at room temperature until the monomer thickened, and then transferred to an oven (37 °C) until the monomer hardened. After the methyl methacrylate was completely hardened, a LeicaSP600 hard tissue slicer (Leica, Wetzlar, Germany) was used to prepare 100-μm-thick tissue sections, which were further polished to 50 µm, sealed, and repolished. We observed the sections and calculated the coverage ratio for all the stents in each section, and Student’s *t* test was used for statistical analysis.

### Evaluation of the toxicity of the nanofiber scaffold mats

The nanofiber scaffold mats were implanted under the abdominal skin of C57 male mice. Tissue specimens were collected 1 month and 3 months after implantation and were stained with H&E to observe the inflammatory reaction of the tissue because of the mats.

### PDGF-BB-induced inflammation and rosuvastatin-treated inflammatory-SMC viability

PDGF-BB was used to establish an inflammatory-SMC model. Briefly, PDGF-BB was dissolved in sterilized H_2_O and diluted with DMEM to 0 ng/mL, 1 ng/mL, 10 ng/mL, 20 ng/mL, 50 ng/mL, 100 ng/mL, and 1000 ng/mL. Rat aortic SMCs were seeded onto 96-well plates at a density of 3 × 10^3^ cells/well in triplicate and cultured in DMEM supplemented with 10% fetal bovine serum (FBS; Epizyme, Beijing, China). Upon reaching 50% to 60% confluence, SMCs were incubated under serum-starvation conditions for 24 h, after which cell viability was measured using the CCK-8 assay, and absorbance was measured at 450 nm using a spectrometer (Thermo Fisher Scientific).

To evaluate rosuvastatin toxicity against contractile SMCs, cells were seeded onto 96-well plates at a density of 3 × 10^3^/well in triplicate and subjected to starvation conditions for 24 h. Rosuvastatin was then dissolved in dimethyl sulfoxide (Sigma-Aldrich) and diluted with DMEM to 0 µM, 0.01 µM, 0.1 µM, 1 µM, 10 µM, 20 µM, and 100 µM. Cell viability was measured with the CCK-8 assay, as previously described.

To determine the effective rosuvastatin concentration for inhibiting PDGF-BB-mediated effects, cells were seeded and starved under the same conditions described previously. After 24 h, cells were treated with a mixed solution of 10 ng/mL PDGF-BB and different doses of rosuvastatin (0, 0.01, 0.1, 1, 5, 10, 20, and 100 µM) for 24 or 48 h, with cell viability measured using the CCK-8 assay.

### Phalloidin assay

SMCs were stimulated with 10 ng/mL PDGF-BB for 24 h, seeded onto nanofiber scaffold mats at a density of 2 × 10^4^/well, and cultured for 48 h. The cultured cells were fixed with 4% PFA overnight and stained with phalloidin solution for 60 min according to manufacturer instructions (Meilun Bio, Dalian, China). Cells were then counterstained with 4′,6-diamidino-2-phenylindole (DAPI; Bioss, Shanghai, China) for 10 min. Similarly, SMCs were seeded onto 24-well plates at a density of 1.5 × 10^4^/well and cultured for 24 h. The cultured SMCs were then starved for 24 h, and the cells were fixed and stained with phalloidin solution (Meilun Bio) and DAPI, as previously described. A confocal fluorescence microscope (Carl Zeiss) was used to observe the cytoskeleton.

### Cell proliferation assay

SMCs were seeded onto 24-well plates at a density of 2 × 10^4^/well in triplicate. The cells were then starved prior to intervention and divided into groups, as follows: controls; SMCs stimulated with 10 ng/mL PDGF-BB (PDGF); SMCs treated with 10 µM Rosuvastatin (Rosu); and SMCs co-treated with 10 ng/mL PDGF-BB and 10 µM rosuvastatin (PDGF + Rosu). Each group was cultured for 24 h, after which an EdU cell proliferation detection kit (Beyotime) was used to evaluate the proliferation rate according to manufacturer instructions. The cells were counterstained with Hoechst-33342 (Beyotime) for 10 min and then observed with a confocal fluorescence microscope (Carl Zeiss). Hoechst-33342 and EdU staining showed blue and green fluorescence, respectively. ImageJ software (National Institutes of Health, Bethesda, MD, USA) was used to count Hoechst-33342- and EdU-labeled cells, and the EdU/Hoechst-33342 ratio was then calculated.

### Scratch test

Rat aortic SMCs were seeded onto six-well plates at a density of 1 × 10^5^/well in triplicate. Upon reaching 80% density, the cells were cultured in serum-free DMEM for 24 h. Pipette tips were then used to scratch the cell monolayer across the center of each well, followed by washing away detached cells with sterile PBS. The cells were divided into control, PDGF, Rosu, and PDGF + Rosu groups, and each group was cultured for 24 h. Three images were photographed for each well and processed using ImageJ software (National Institutes of Health). The areas in the images where cells remained after 24 h were compared.

### Transwell tests

SMCs were seeded onto a 12-well plate at a density of 5 × 10^4^/well in triplicate and starved prior to intervention. The cells were then digested and seeded into 8-µm-pore Transwell chambers (Corning, Corning, NY, USA) at a density of 1 × 10^4^/well, followed by addition of 600 µL of DMEM containing 10% FBS to the lower chamber and incubation at 37 °C for 6 h. The medium in the upper chambers was then replaced with serum-free DMEM and incubated for another 18 h. The cells were then fixed with 4% PFA for 30 min at room temperature and then with crystal violet (Beyotime) for 5 min. Cells attached to the interior of the upper chamber were removed with a cotton swab, and the chambers were placed on a new 24-well plate and air-dried for 15 min. The number of cells on the surface of the lower chamber was calculated and photographed under a microscope.

### Apoptosis of rosuvastatin-treated contractile SMCs

An Annexin V-fluorescein isothiocyanate (FITC) cell apoptosis detection kit (Beyotime) was used to detect the effect of rosuvastatin on contractile SMCs according to manufacturer instructions. A flow cytometer (BD Biosciences, Franklin Lakes, NJ USA) was used to detect Annexin V-FITC and propidium iodide staining (green and red fluorescence, respectively).

### RNA extraction and real-time PCR

RNA was isolated using an RNA purification kit (Yishan, China) according to manufacturer instructions, and RNA integrity was quantified using a NanoDrop 1000 spectrophotometer (Thermo Fisher Scientific). Reverse transcription was performed using a fast all-in-one RT kit (Yishan, China) according to manufacturer instructions. Real-time PCR was performed using Hieff qPCR SYBR Green master mix (Yeasen, China) and detected with a real-time PCR system (7900HT; Applied Biosystems, Foster City, CA, USA). No nonspecific amplification was observed based on the dissociation curve. We used glyceraldehyde 3-phosphate dehydrogenase (*GAPDH*; Sangon Biotech, Shanghai, China) as an internal control. Data were analyzed using the 2^−ΔΔCT^ method and expressed as fold change relative to the respective control. Primer sequences are listed in Additional file 1: Table S1.

### Western blot

Proteins were removed from rat aortic SMCs following lysis and after intervention. The cells were divided into four groups, as described, and 40 µg of protein per lane was separated using 10% sodium dodecyl sulfate-polyacrylamide gel electrophoresis (Epizyme, Beijing, China), transferred to a polyvinylidene difluoride membrane (Millipore Sigma, Billerica, MA, USA), and blocked with western quick-blocking buffer (Beyotime) for 15 min at room temperature. The blocked membranes were then incubated overnight with primary antibodies, namely goat anti-SM22α (1:500; Abcam, Cambridge, UK), rabbit anti-OPN (1:1000; Abcam), and rabbit anti-MMP9 (1:1000; Abcam). After 12 h to 14 h, the membranes were incubated with a horseradish-peroxidase-conjugated secondary antibody (HuaBio, Hangzhou, China) for 1 h at room temperature. The immunoblots were probed using an enhanced chemiluminescence substrate (Thermo Fisher Scientific), and an imaging system (Bio-Rad Laboratories, Hercules, CA, USA) was used for blot detection and recoding of chemiluminescence. The results were normalized to that of GAPDH, and experiments were performed in triplicate.

### Cytokine and chemokine analyses

SMCs were seeded onto a 24-well plate containing control, Rosu 50, Rosu 75, and Rosu 100 nanofiber mats at a density of 1 × 10^4^/well. The cells were cultured for 48 h, and cellular supernatants were collected and used for IL-1β, IL-6, MCP-1, TNF-α, vascular endothelial growth factor, fractalkine (CX3CL1), and RANTES (CCL5) testing with a MILLIPLEX MAP rat cytokine/chemokine factor panel (Millipore Sigma). Similarly, SMCs were transferred onto a 24-well plate at a density of 2 × 10^4^ cells/well, followed by treatment as previously described in this section, with DMEM was used as a control. At 24 h, the culture supernatants were collected and tested for IL-1β, IL-6, MCP-1, and TNF-α levels.

## Supplementary Information

**Additional file 1: Figure S1.** Cell viabilities of SMCs cultured for 24 h with different concentrations of (A) PDGF-BB-induced synthetic SMCs, (B) rosuvastatin-treated contractile SMCs, and (C) rosuvastatin-treated synthetic SMCs. **Figure S2.** Cell apoptosis of rosuvastatin-treated contractile SMCs. **Table S1.** mRNA sequnce

## Data Availability

The datasets used and/or analyzed are available from the corresponding author upon reasonable request.

## Abbreviations

P(LLA-CL): Poly(L-lactide-*co*-caprolactone)
PDGF-BB: Platelet-derived growth factor-BB
IA: Intracranial aneurysm
DES: Drug-eluting stents
SMC: Smooth-muscle cell
VSMC: Vascular smooth-muscle cell
EC: Endothelial cell
PCR: Polymerase chain reaction
PTFE: Polytetrafluoroethylene
TEM: Transmission electron microscopy
SEM: Scanning electron microscopy
PFA: Paraformaldehyde
CCK-8: Cell counting kit-8
H&E: Hematoxylin and eosin
FBS: Fetal bovine serum
DMEM: Dulbecco’s modified Eagle medium
DAPI: 4′,6-Diamidino-2-phenylindole
IL: Interleukin-1β
FITC: Fluorescein isothiocyanate
MCP-1: Monocyte chemoattractant protein-1
OPN: Osteopontin
SMA: Smooth muscle actin
TNF-α: Tumor necrosis factor-α
VEGF: Vascular endothelial growth factor
GAPDH: Glyceraldehyde 3-phosphate dehydrogenase
CCA: Common carotid artery
DSA: Digital subtraction angiography
