# Supplementary material for: A heparin–rosuvastatin-loaded P(LLA-CL) nanofiber-covered stent inhibits inflammatory smooth-muscle cell viability to reduce in-stent stenosis and thrombosis
Source: J Nanobiotechnology. 2021 Apr 29;19:123. doi: 10.1186/s12951-021-00867-8 (PMC8086342; doi:10.1186/s12951-021-00867-8)
Supplement: Supplementary file 1 — Additional file 1: Figure S1. Cell viabilities of SMCs cultured for 24 h with different concentrations of (A) PDGF-BB-induced synthetic SMCs, (B) rosuvastatin-treated contractile SMCs, and (C) rosuvastatin-treated synthetic SMCs. Figure S2. Cell apoptosis of rosuvastatin-treated contractile SMCs. Table S1. mRNA sequnce [file 12951_2021_867_MOESM1_ESM.docx]

Additional File

| 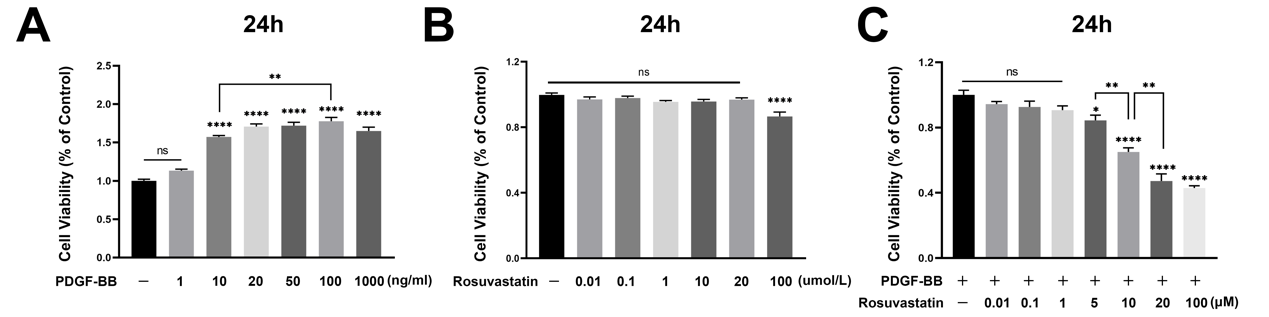 |
| --- |
| **Figure S1.** Cell viabilities of SMCs cultured for 24 h with different concentrations of (A) PDGF-BB-induced synthetic SMCs, (B) rosuvastatin-treated contractile SMCs, and (C) rosuvastatin-treated synthetic SMCs.   \| 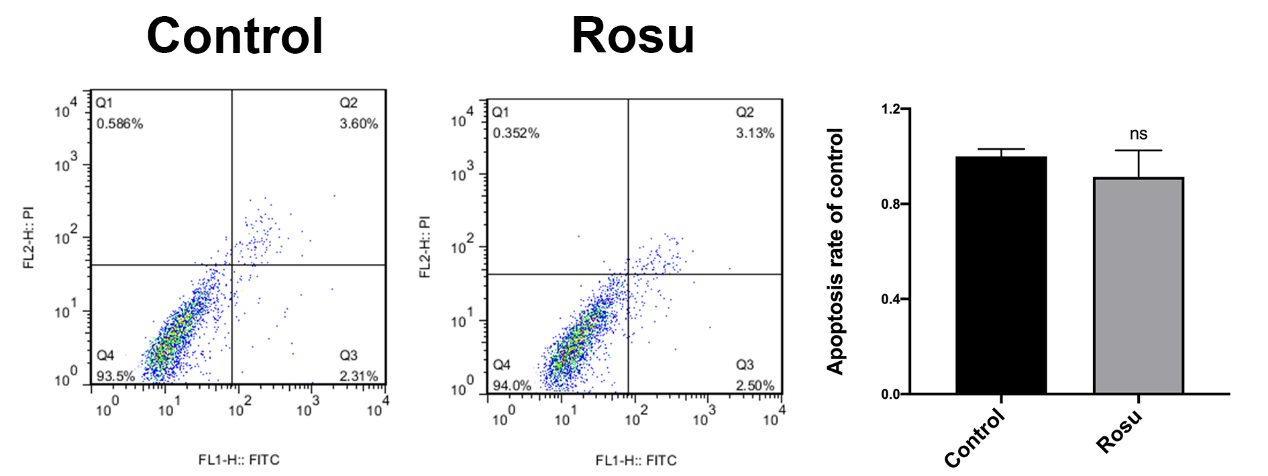 \| \| --- \| \| **Figure S2.** Cell apoptosis of rosuvastatin-treated contractile SMCs. \| |

| Primers | 5’-3’ | Sequence |
| --- | --- | --- |
| $\alpha$-SMA | Forward | TGGAAAAGATCTGGCACCAC |
|  | Reverse | TCCGTTAGCAAGGTCGGATG |
| SM22-$\alpha$ | Forward | ATGGCCAACAAGGGTCCATC |
|  | Reverse | AACTTGCTCAGAATCACGCCA |
| OPN | Forward | CCAGCCAAGGACCAACTACA |
|  | Reverse | CTGCCAAACTCAGCCACTTTC |
| TNF-$\alpha$ | Forward | GTGATCGGTCCCAACAAGGA |
|  | Reverse | CGCTTGGTGGTTTGCTACG |
| MCP-1 | Forward | TCCACCACTATGCAGGTCTC |
|  | Reverse | CATTAACTGCATCTGGCTGAG |
| MMP-2 | Forward | CAAGCCCAAGTGGGACAAGA |
|  | Reverse | CCATGCTCCCATCGACCAAA |
| MMP-9 | Forward | CAAGGACGGTCGGTATTGGA |
|  | Reverse | CGTGCGGGCAATAAGAAAGG |
| GAPDH | Forward | AGTGCCAGCCTCGTCTCATA |
|  | Reverse | TGAACTTGCCGTGGGTAGAG |
| **Table S1.** mRNA sequnce | | |
